# Residential secondhand smoke in a densely populated urban setting: a qualitative exploration of psychosocial impacts, views and experiences

**DOI:** 10.1186/s12889-022-13561-7

**Published:** 2022-06-11

**Authors:** Grace Ping Ping Tan, Odelia Teo, Yvette van der Eijk

**Affiliations:** Saw Swee Hock School of Public Health, National University of Singapore, MD1 Tahir Foundation Building 12 Science Drive 2 #09-01C, 117549 Singapore, Singapore

**Keywords:** Air pollution, Environment, Home, Psychosocial, Secondhand smoke, Smoking, Tobacco

## Abstract

**Background:**

People remain exposed to secondhand smoke, a serious health hazard, inside their home as households face challenges in setting no-smoking rules or are exposed to secondhand smoke drifting in from neighbouring homes. This study explores the psychosocial impacts, views, and experiences with residential secondhand smoke in a densely populated urban setting.

**Methods:**

In-depth online or face to face interviews with 18 key informants who had been involved in public discourse, policy, advocacy or handling complaints related to residential secondhand smoke, 14 smokers, and 16 non-smokers exposed to secondhand smoke inside their home. All participants were residents of Singapore, a densely populated, multi-ethnic city-state. Interview transcripts were coded in NVivo using a deductive and inductive coding process.

**Findings:**

Secondhand smoke has wide-reaching impacts on physical and psychosocial wellbeing, even if smokers tried to minimise secondhand smoke. Feelings of anxiety and stress are generally tied to feeling discomfort in one’s personal space, a perceived lack of control over the situation, resentment towards smokers, and concerns over the health effects. Family, community, and cultural dynamics add complexities to tackling the issue, especially in patriarchal households. Secondhand smoke exposure from neighbours is considered a widespread issue, exacerbated by structural factors such as building layout and the COVID-19 pandemic. Resolving the issue amicably is considered challenging due to the absence of regulations and a reluctance to stir up conflict with neighbours. While smokers took measures to reduce secondhand smoke, these were described as ineffective by other participants. Smokers appeared to have contrasting views from other participants on what it means to smoke in a socially responsible manner.

**Conclusion:**

Given the wide-reaching psychosocial impacts of residential secondhand smoke, there is a case for stronger interventions, especially in densely populated urban settings where it is more difficult to avoid.

**Supplementary Information:**

The online version contains supplementary material available at 10.1186/s12889-022-13561-7.

## Background

Secondhand smoke (SHS), a toxic mix of over 7,000 harmful chemicals, kills 1.2 million non-smokers each year [1]. It is harmful even at low levels [2–4], especially to children or people with pre-existing health conditions [5–8]. Although well-enforced smokefree legislations protect people from SHS in public places, people often remain exposed to SHS inside their homes [9, 10]. In-home SHS exposure increases the risk of cardiovascular and respiratory diseases, lung cancer and asthma, [11–14] and is associated with poorer mental health outcomes in adults [15] and children [16]. It has also been associated with depression, [17–22] stress, [23, 24] and anxiety [22, 25]. Psychosocial issues related to home SHS exposure have also been reported in the literature. These include family strife and unhappiness, [26] the need to navigate social and relational norms, [27] heightened distress and lowered sense of agency among caregivers of higher risk children, [28] a sense that one’s privacy has been invaded and the ability to enjoy one’s home has been undermined [29], and the tension residents face between sympathizing with the neighbour’s smoking addiction and the belief in a collective responsibility to refrain from actions detrimental to fellow residents [30].

As there is no risk-free exposure to SHS, and air purifiers and ventilation are ineffective protection mechanisms, [31–33] the only way to protect people from SHS is to eliminate smoking where others are exposed [34]. However, households often face challenges in agreeing on a smoking ban and end up compromising on less effective strategies such as restricting smoking to specific parts of the home or smoking out of a window [35–37]. Even non-smoking households are exposed to SHS from neighbouring homes, especially those living in multiunit housing [38–41]. A Hong Kong study found that, among non-smoking adolescents, those exposed to SHS from neighbours were more likely to report symptoms of respiratory disease [42]. Although no country has banned smoking inside homes, patchwork legislations exist in the United States covering public multiunit housing, [43] multiunit housing in 67 Californian municipalities, [44] and apartment complexes with voluntarily adopted smokefree measures [45, 46].

In Singapore, a city-state in Southeast Asia with a multi-ethnic (predominantly Chinese, Malay and Indian) population, the issue of SHS exposure in homes has been the subject of Parliamentary debates since 2017 due to high volumes of complaints about SHS drifting in from neighbouring homes, especially following the COVID-19 lockdown measures [47, 48]. In a 2020 survey, 85% of Singapore residents supported a proposal to ban smoking near a window or balcony in multiunit housing [49]. With 95% of Singapore residents living in multiunit housing, including condomiums and public housing estates [50], SHS drift into others’ homes appears to be widespread despite a low adult smoking prevalence at 11% (17% in males, 3% in females) [51]. Comprehensive smokefree legislations cover many public places and shared residential spaces such as common corridors, stairwells and void decks (the communal spaces on the ground floor of public housing blocks) [52, 53]. Although the Singapore Government has not articulated any formal plans to regulate smoking inside homes, public and Parliamentary debates were ongoing as at September 2021 [54].

Little is known on the nuances of how residential SHS affects individuals, families and neighbours living in densely populated, multi-ethnic urban settings such as Singapore. This study aims to understand the psychosocial impacts, views and experiences of residential SHS exposure in a densely populated urban setting.

## Methods

In March-August 2021, we conducted in-depth interviews with 18 key informants who had been involved in public discourse, policy, advocacy, or handling complaints related to residential SHS and 30 Singapore residents (Citizen or Permanent Resident) who smoked in their home or were exposed to SHS in their home (Table 1). We recruited key informants via email invitation, and residents via email flyers, social media and snowball recruitment. Prior to joining the study, residents provided information on their sociodemographics, smoking habits and history of residential SHS exposure to enable sample balancing in terms of age, gender, ethnicity, housing type, smoking status, and experiences with in-home SHS.

**Table 1:** Details of interviewees

**Residents**	**N (%)**
Age	
20 – 29	12 (40.0)
30 – 39	9 (30.0)
40 – 49	4 (13.3)
50 +	5 (16.7)
Gender	
Female	15 (50.0)
Male	15 (50.0)
Ethnicity	
Chinese	19 (63.3)
Malay	4 (13.3)
Indian	4 (13.3)
Other	3 (10.0)
Current housing	
HDB (public housing)	26 (86.7)
Condominium	3 (10.0)
Dormitory	1 (3.3)
Smoking status	
Current smoker	14 (46.7)
Non-smoker	16 (53.3)
SHS exposure at home (non-smokers only)	
Household member smokes at home	12 (75.0)
No household members smoking at home	3 (25.0)
Frequency of exposure to neighbour’s SHS	
Daily	15 (50.0)
Non-daily	9 (30.0)
Not at all	6 (20.0)
**Key informants**	**N (%)**
Role	
Academic	2 (11.1)
Advocate	4 (22.2)
Condo Management	2 (11.1)
Doctor	3 (16.7)
Legal Expert	2 (11.1)
Policymaker	4 (22.2)
Public Officer	1 (5.6)

Interviews with key informants were one-on-one while interviews with residents were one-on-one or dyadic, in cases where two household members preferred to be interviewed together. Interviews lasted 40–70 min each, were conducted in English, the most widely spoken language in Singapore, and were done either face to face (*n* = 1) or online (*n* = 47) using Zoom conferencing. Interview questions followed an open-ended format (Table 2). We reimbursed each resident or dyad with S$50 cash.

**Table 2:** Interview guide for residents and key informants

**Residents**
Background information
1. Please tell me about the people you live with and your/their smoking habits
2. Please tell me about your experiences with secondhand smoke at home
3. Do you think secondhand smoke has affected you or your family? Please describe
4. Do you think SHS affects health or wellbeing in any way? Please describe
Questions for households with a smoker
5. Do the smoker or other household members ever try to minimise the SHS in the home?
6. How do the household members feel about the person’s smoking habit?
7. Have neighbours ever approached you or anyone in your household about secondhand smoke? Please describe your experience
8. What are your views on socially responsible smoking in the residential setting?
Questions for residents affected by SHS drift from neighbours
9. Please describe your experience of SHS going into your home from other units
10. How do you minimise SHS from your neighbours going into your home? Does it work?
11. Have you tried approaching your smoking neighbours about the issue? Why (not)?
12. Are there other ways in which you tried to solve the issue? What was your experience?
13. What are your views on socially responsible smoking in the residential setting?
**Key informants**
1. Please tell me more about your experiences with the residential secondhand smoke issue
2. What are your views on this issue? How do you think it affects people?
3. Do you think the residential SHS issue has changed following the COVID-19 pandemic?
4. How do people deal with the issue of residential SHS? What are your views on this?
5. What are your views on socially responsible smoking in the residential setting?

Interviews were audio recorded, transcribed verbatim, and imported into NVivo. We developed an initial codebook with deductive codes originating from a priori topics in the interview guides, and subsequently modified the codebook to include inductive codes upon multiple reading of the transcripts. Finally, all transcripts were double coded and compared among the researchers working independently to ensure coding consistency. Similar codes were combined and new codes were added to the codebook during the coding process. Discrepancies were reviewed and discussed by the researchers until consensus was reached. Codes were then organised into categories, sub-categories and overarching themes (see supplement for codebook). Although data for all participants were coded together, we distinguished between key informants, smokers, and non-smoking residents to identify differences in the themes emerging from each group.

The study was approved by the National University of Singapore Institutional Review Board (reference NUS-IRB-2021–79). Participants were informed of the study procedures and risks and provided written informed consent prior to the interview.

## Results

Participants discussed four themes: (1) their perceptions and experiences with residential SHS; (2) strategies used to minimise SHS exposure from neighbours; (3) strategies used to minimise SHS exposure from smokers in the household; and (4) views on what it means to smoke in a socially responsible manner. In what follows, we discuss findings from key informants, smokers and non-smoking residents, with quotes to illustrate our points where relevant.

### Perceptions and experiences with secondhand smoke

#### Harm perceptions of secondhand smoke

Participants unanimously agreed that SHS is a serious health hazard, especially to vulnerable people such as children, associating it with lung cancer, asthma and cardiovascular diseases. Some participants knew someone who had died from SHS exposure. However, participants appeared to have various misperceptions on the relative harms of SHS. A few, including smokers, described SHS as more harmful than active smoking, while others believed that SHS is only harmful if exposure is heavy or prolonged:

That kind of low level, I guess is OK, I guess it's not so bad. Because your lungs… rest and doesn’t collect all these particles all the time. – Resident (Smoker)

Others, especially key informants, were unsure and highlighted a need for more evidence on the dose–response effect of SHS exposure:

There is a need to show in concrete terms the harm that SHS poses… exactly when, and at what levels and how, does exposure to SHS become harmful? – Public Officer

Several participants believed that the harmfulness of SHS depends on other factors such as genetics or pre-existing conditions:

…someone with asthma or some respiratory issues, it would be different. Smaller amounts could be an immediate reaction and all of that, but like seeing regular, healthy able-bodied, and stuff, I don't imagine it's that bad or maybe I'm just being hopeful. – Resident (Smoker)

#### In-home secondhand smoke from neighbours

Key informants described SHS incursion from neighbours as a longstanding and common issue, affecting many residents due to the high density in which people in Singapore live. Key informants and residents also described a building’s layout, airflow, and proximity to areas where people smoke (e.g. stairwells and common corridors), as reasons why some homes may be more affected than others:

My room is at the back side, so if they go to the back side, can smell it from there, if they smoke at the front of the HDB flat (public MUH), my living room, I can smell it. – Resident (Non-Smoker)

Participants described the SHS from neighbours as difficult or impossible to escape:

I’ve lived in three different condos in Singapore… In all three, I had smoker neighbours. It's not like moving house can solve this problem. Right now it's worse in this current condo that I'm staying in. We were sandwiched among three smokers, upstairs, downstairs and next-door. – Advocate

#### Secondhand smoke in common residential areas

Participants commonly recalled experiences with people smoking in common residential spaces where smoking is prohibited such as void decks, common corridors and stairwells. Most smokers admitted to smoking in these areas, out of convenience or a perceived lack of enforcement:

Let’s just put it down to pure laziness, so that’s why I smoke at the common corridor and staircase landing. – Resident (Smoker)

As long as you don’t get caught, it’s not a problem. So I have seen people smoking at the void decks, I have seen people smoking in the corridors… we do it, but we do it discreetly. – Resident (Smoker)

Smokers who had observed enforcement of this smoking ban felt that this was an effective deterrent, while others were unsure of the rules:

I generally follow it because I also don't want to just randomly get fined $300 by plainclothes NEA (National Environment Agency) officer. – Resident (Smoker)

…at our void deck, where the rubbish bin is, there is the smoke thing [rubbish bin with ashtray]. So I think it's okay to smoke there. – Resident (Smoker)

#### Secondhand smoke exposure following the COVID-19 pandemic

Both key informants and residents felt that residential SHS had increased following the COVID-19 pandemic, primarily due to people spending more time at home. However, a few residents living with smokers felt that the pandemic had either improved or not changed their exposure to SHS, in cases where habits of the smokers they lived with remained the same or the lockdowns resulted in them not living together with the smoker.

#### Personal impacts of secondhand smoke

When asked about how SHS exposure has affected them, participants reported a wide range of medical conditions. Those exposed to SHS by people smoking inside their home recalled experiences with lung cancer, breast cancer, asthma and eye conditions. Even those whose family members only smoked in confined or outdoor parts of the home reported sinus and respiratory issues such as chest pains and breathlessness. Participants from non-smoking households reported respiratory symptoms, worsening of their asthma, allergic reactions, headaches and migraines following SHS incursion from neighbours. They also described medical conditions in children, notably respiratory issues, sinus issues and eye irritation, as being caused or aggravated by a neighbour’s SHS:

The poor toddler has been suffering from chronic bronchitis and even pneumonia due to her downstairs chain-smoker neighbour. – Advocate

Participants also reported negative impacts on their mental wellbeing, with SHS described as a source of stress, anxiety, negative moods, and sleeping disorders. Those exposed to SHS from neighbouring units commonly indicated these as being tied to a sense of frustration, hopelessness, and constant worrying about the health effects:

My wife is so stressed, she can’t sleep. Every night she’s got to check on the children, see whether they’re okay. – Advocate

…it is distressing because you are in your own home, you expect to have quiet enjoyment of your own home… it feels like you are being suffocated by the smoke, then you keep thinking about the health effects that you might be experiencing. – Resident (Non-Smoker)

Participants highlighted the inconvenience and frustration of constantly having to close windows to block SHS from neighbouring homes. One participant, whose mother was a cancer patient, described how continually having to open and close windows was disruptive to her recovery:

…she wants fresh air. Open window then got smoke, to her is troublesome also because when she’s resting, after 5 minutes while she is lying in the bed so comfortable, she have to get up to close the window. – Resident (Non-Smoker)

Participants described how having to keep their windows closed made the home stuffy and unhygienic in Singapore’s hot and humid climate:

My toilet floor is always wet and because when it's wet and it's moist, it affects my walls and windows with mould and mildew. – Resident (Non-Smoker)

I can’t even smell fresh air in my own personal space. – Resident (Non-Smoker).

Participants reported feeling nauseous, irritated, or frustrated by the SHS smell. This was a theme even among most of the smokers:

As a smoker, I hate secondhand smoke. I don’t like the smell. – Resident (Smoker).

I like to smoke my one stick, I don’t want to smell the smoke of everybody else… the smell of cigarettes that's not the one that you’re inhaling is different… you’re getting like the ash at that point, rather than the nice nicotine. – Resident (Smoker)

Participants also highlighted the inconvenience and financial burden of having to re-wash laundry that had been exposed to SHS or ash dropping from neighbouring units and having to run airconditioning instead of opening a window.

### Minimising SHS from neighbours

#### Strategies to minimise secondhand smoke from neighbours

When faced with SHS from neighbours, most affected participants reported that they close their windows or doors to block out the SHS. This was generally considered the most effective strategy, although it came at the cost of forfeiting fresh air and ventilation in their homes:

We don’t have aircon in the house, so we depend a lot on fan and we do need ventilation some way, somehow. – Resident (Non-Smoker)

Some participants also reported using an air purifier or fan, or moving into another room to avoid the SHS:

I’ve tried air purifier before. It doesn’t work because it’s not fast enough. – Resident (Non-Smoker)

So, the moment I detect the smoke I quickly tell them [children], ‘hey, there’s somebody smoking, you all go to your room, close the door..’ – Resident (Non-Smoker)

#### Confronting neighbours about secondhand smoke

A few participants had confronted their neighbours about SHS, with approaches ranging from friendly to antagonistic. Some had approached neighbours with gifts, a polite note on the door, or a friendly conversation emphasizing the impact of SHS on their children’s health:

I thought in the first place, we could address it quickly in a sense that I pay him a visit and still talk to him nicely, saying that can you close the window and not allow your smoke [to] drift into my place? – Advocate

More antagonistic approaches included leaving notes in common areas singling out units suspected as the SHS source, scolding the neighbours, or spraying insecticide on them:

She will go upstairs and like scold the person and be like ‘look at my clean clothes now.’ – Resident (Non-Smoker)

Last time we stay in HDB [public housing] flat, 4^th^ level, the 5^th^ level always complain. And they spray Baygon [insecticide], spray down. – Resident (Non-Smoker)

Most participants, however, were reluctant to confront their neighbour about SHS as they felt anxious it would lead to conflict or believed that, with no regulations, these efforts would be futile:

I don’t want to actually confront them because that would put me in a difficult position… what if the person gets aggressive? – Resident (Non-Smoker)

…with no regulation for smoking in the house, there’s technically nothing that we as neighbours who are non-smokers can do about it. – Resident (Non-Smoker)

#### Smokers’ responses to neighbour confrontation

Regardless of the approach, most attempts to settle the issue directly with neighbours were described as unsuccessful. The smoking neighbour’s responses ranged from avoidant to hostile, while others responded amicably but took no action to reduce SHS:

They kind of just nod their head and then walk away. – Resident (Non-Smoker).

[The] downstairs smoker refused to open the door on multiple occasions. The upstairs smoker insisted that it's his right to smoke at home, because it's not against the law and told us to mind our own business. Then the next-door neighbour turned aggressive. – Advocate

Smokers or their family members, when asked how they would respond to a neighbour’s request to reduce SHS, gave a range of responses ranging from reluctant to willing to compromise. Those who were reluctant believed that their SHS was unlikely to affect others or that they were entitled to smoke in their home:

I will get a bit defensive because this is my house. – Resident (Smoker).

I will tell them to close their own windows. Because I actually smoke in the middle of my living room so, I have no idea how my secondhand smoke will actually affect them. – Resident (Smoker)

Those who were willing to compromise generally had more awareness of the health effects of SHS and expressed a stronger interest in keeping a good relationship with neighbours:

I would apologize first because I would feel really, really bad about it since I'm very cautious about this kind of stuff, honestly. – Resident (Smoker)

…we don’t want any trouble with our neighbours. We have a very good relationship with our neighbours. – Resident (Smoker)

Other smokers indicated that their response depended on the neighbour. Those perceived as inconsiderate or unfriendly were more likely to be met with reluctance. A few smokers indicated they might be more sympathetic towards those experiencing health issues:

I will only stop if you stop, stop stomping and moving furniture in the middle of the night. – Resident (Smoker)

It really depends on how belligerent they are about it, to be honest… If someone’s bringing up health complications, then I would be a lot more understanding. – Resident (Smoker)

For smokers, the main reason they did not smoke inside their home with windows closed was to minimise SHS exposure to their family members, especially children:

He’ll run to the kitchen window and smoke, which I think is not nice to the other neighbours but you can see he’s trying to be considerate for his grandchildren and his guests. – Resident (Non-Smoker)

While most smokers were reluctant to smoke outside their home due to the inconvenience, in one case it was more challenging as the smoker had a mobility issue:

He’s got some mobility issues, some health condition… for him specifically to go down and smoke and taking our time (to take him down), I think it’s a bit, it’s pretty hard for us. That’s why we allow him to smoke in the house instead. – Resident (Non-Smoker)

### Minimising secondhand smoke from smokers in the household

#### Smokers’ strategies to minimise secondhand smoke

The strategy most commonly taken by smokers to minimize SHS in the home was to limit where in the home they smoke, usually to an outdoor area (e.g. balcony) or enclosed space within the home (e.g. bathroom or private room). The majority also closed doors to minimize SHS incursion from these spaces into other parts of the home. Another commonly reported strategy was to smoke near a window or ventilation system, such as an air filter or fan, to blow out the smoke. Two participants also avoided smoking inside the home unless their family members were out.

#### Non-smokers’ strategies to minimise secondhand smoke

Strategies most reported by non-smokers to minimize in-home SHS were closing doors and using fans or air purifiers. They generally described these strategies as ineffective:

Ultimately I still can smell it. Like no matter what, if he’s smoking I can smell it. – Resident (Non-Smoker)

A few participants reported avoiding the areas where household members smoke:

I got fed up, I spend all my time in the [bed]room. – Resident (Non-Smoker).

#### Confronting smokers about secondhand smoke in the home

Non-smokers described various approaches they had used to persuade a family member to reduce in-home SHS. Some participants simply set a no-smoking house rule or asked the family member to restrict their smoking to specific areas within the home. Others had attempted to talk to the smoker about quitting, but with little success. A minority had approached the matter from a health perspective, but reported that they had little success with this unless they were able to make a personal appeal:

The facts are useless in a scenario like this… I can say to him now, ‘you know my brother has cancer right? So you probably shouldn’t smoke around him.’ Then he'll get it because then he has a personal connection to the matter. – Resident (Non-Smoker)

Others simply expressed their discomfort or disapproval when the family member smoked inside the home, in various ways:

…being sarcastic, like cough in front of them when they smoke. – Resident (Non-Smoker)

I’m very angry, I scream at him. – Resident (Non-Smoker).

A few smokers were described as willing to change their habits after pressure from family members, while others were described as reluctant to change. This reluctance was often expressed as an unwillingness to listen:

It was quite clear that he was not receptive. Kind of, in one ear, out the other. – Resident (Non-Smoker)

I always say, ‘can you at least do it outside?’ But he’ll just be like, ‘yah lah, ya lah, ya lah’. But he doesn’t do it. – Resident (Non-Smoker)

#### Issues in addressing in-home secondhand smoke

The inability to resolve the in-home SHS issue was described as a source of conflict within some families and guilt among smokers:

I get anger [sic] that, wah, you just don’t bother, you’re just enjoying yourself smoking. You don’t care about me, a non-smoker, inhaling all this. – Resident (Non-Smoker)

I know it’s not good, especially for my children. So sometimes I actually feel sad that I can’t quit. – Resident (Smoker)

When asked about barriers to getting smokers to reduce in-home SHS, participants described how, since smoking had become a deeply ingrained habit, it was difficult to change their smoking routine. They also cited the inconvenience of having to move outside to smoke. Traditional patriarchal norms made it difficult for some participants to confront family members, especially in cases where the smoker was their father:

You don’t own the house. You do not dictate to him. I mean, he has, you know, Asian society, he’s still the senior. – Resident (Non-Smoker)

### Views on socially responsible smoking

While participants, especially smokers, held the view that people are entitled to smoke or do what they like inside their own property, they also strongly felt that smokers don’t have a right to smoke in their homes if it affects others. These views were echoed equally among smokers, non-smokers and key informants:

What about the right to throw garbage out of the house? What about the right to pour water out of your house… do we allow for those rights? – Academic

Neighbours or people who live in their house and say, ‘it’s my house, my own problem, I smoke, my own problem. I don’t disturb you’, which is a very wrong concept they have, because definitely they’re bothering somebody. – Resident (Non-Smoker)

Key informants, non-smokers and some smokers generally described smokers as being on a spectrum, ranging from those who voluntarily try to smoke in a socially responsible manner to those who appear to be indifferent or unaware of their impact on others:

I got a handful of smoker friends, they are very considerate… but there are also [an] inconsiderate group that we are facing. – Advocate

Several participants felt that Singapore’s culture of entitlement compounds the issue by encouraging selfishness and disregard for others among some smokers:

We’re just generally very entitled people. We don’t really care about other people. – Resident (Non-Smoker)

Most smokers described themselves as taking steps to smoke in a socially responsible manner. However, they appeared to have differing views on what that entails in practice. While some smokers went to great lengths to avoid smoking near people, especially children, others simply complied with no-smoking rules. In general, younger and female participants perceived SHS as more harmful and expressed a greater desire to smoke in a socially responsible manner. A few participants described specific actions that had been taken to minimize SHS disturbances to their neighbours, including seeking consent from the neighbours to smoke or voluntarily smoking in an area downstairs, away from the building.

## Discussion

This is, to our knowledge, the first study detailing the psychosocial aspects of residential SHS in Singapore, and the first in Southeast Asia to also explore SHS incursion from neighbouring homes and include the perspectives of smokers and other stakeholders. Residential SHS has wide-reaching impacts on physical and psychosocial wellbeing, even if smokers try to minimise SHS or if the SHS is from a neighbouring home. This is consistent with literature demonstrating the adverse health impacts of low levels of SHS exposure, [55–58] as well as evidence associating SHS exposure with mental health conditions such as depression, [17–22] stress, [23, 24] and anxiety [22, 25]. Our findings indicate that these negative mental impacts are tied to a sense of entrapment or discomfort in one’s personal space, a perceived lack of control over the situation, resentment or frustration towards smokers, and constant worrying about the health effects, especially on children. For smokers, inability to resolve the issue was a source of guilt and family conflict. Residential SHS may have more of a psychosocial impact as it encroaches into the private space and is often tied to interpersonal relationships with family members or neighbours [59].

Family, community, and cultural dynamics add further complexity to the problem. In our Singapore households, as well as those in other studies, creating a smokefree norm at home posed interpersonal, structural and cultural challenges, [60, 61] influenced by knowledge and risk perceptions of SHS, one’s sense of agency, interpersonal relationships, and wider community norms [27]. Traditional patriarchal households, as often found in Asian societies, may face additional barriers if the smoker, often a male adult, resists influence from family members. Some of our participants who lived with a smoking husband, brother or father reported this problem, expressing that it would be inappropriate to objecting to the ‘elder’, ‘head of the household’ or the homeowner (roles that are usually held by the husband, brother or father) from doing as he pleases in the home. This has similarly been reported in other studies [27].

Studies from countries with similar patriarchal norms suggest that equipping non-smokers with skills to influence the smoker may help to break down such cultural barriers [62, 63]. Patriarchal norms may also be used to facilitate the creation of smoke-free homes. An emphasis on the role of men as protectors of the family/community, coupled with education on the harms of SHS and smoke-free norms at the societal level, frames the establishment of a smoke-free home as an act of male responsibility in protecting and caring for women and children [64, 65]. This is consistent with our results where male smokers report feeling guilt over exposing their family to SHS knowing that it harms their family members.

Participants described SHS incursion from neighbours as a widespread issue, with building layout, airflow, a unit’s location, and lifestyle factors, such as spending more time at home following the COVID-19 pandemic, cited as factors affecting SHS concentrations. An increase in cigarette-use or SHS exposure at home during COVID-19 lockdown measures have also been reported in other countries [66–70], and the substantial impact on children in smoking households who spent less time in smoke-free places (e.g. school, outdoors) and more time at home has been highlighted [71]. Singapore has a high population density, with 95% of residents living in multiunit housing [50]. Even with a low smoking prevalence, SHS may be more concentrated in crowded urban settings such as Singapore’s, as high rates of in-home SHS have been reported in other densely populated cities including New York City, [72] Los Angeles, [73] and Seoul [74]. In such settings, factors such as building layout, airflow and social distancing measures may have more tangible impacts on SHS levels inside homes. To address the issue of residential SHS, smoking has been banned in public multiunit housing in the United States, [43] and public and private multiunit housing in California [44] and Canada [46, 75, 76] although non-compliance and weak enforcement presented a challenge [77–80]. In Singapore, debates have been ongoing over whether to implement a similar ban, [47, 48, 54] and interventions such as designated smoking points [81, 82] and public education campaigns [83] are being considered.

Due to Singapore’s hot and humid climate, closing the window to block out a neighbour’s SHS was considered unfeasible, leaving participants with neighbourly confrontation as the only recourse. Most were reluctant to do so, expressing a fear of conflict or sense of futility in the absence of regulations. The overall reluctance to approach neighbours may reflect Singapore’s culture, which tends to favour top-down regulation and social harmony over direct confrontation. As in other conflict-averse societies, the ability to resolve the issue amicably may depend on the neighbourly relationship [84]. Our findings suggest that a smoker’s willingness to compromise may also be influenced by their harm perception of SHS and view on what it means to smoke in a socially responsible manner. For some, this meant avoiding exposing others as much as possible while for others it simply meant not breaking the law. While smokers took measures to reduce SHS, these were described as ineffective by non-smokers.

This suggests that public education campaigns may be an effective intervention, if they emphasise that even low SHS levels are harmful and that the only way to smoke responsibly is to completely avoid exposure to others. An approach akin to that of a community-based intervention that was successfully implemented in India [85] and Indonesia [86] might also prove promising for Singapore. The intervention messaged the importance of smokefree environments as a women and children’s health issue, and established smokefree homes as a norm at the community-level [85, 86]. Such an approach may be an effective intervention for protecting people against SHS in their homes in a context where neighbours’ SHS is viewed more as a nuisance than a health threat and addressing SHS incursion at the individual level is too confrontational and daunting.

### Strengths and limitations

Our study design gave participants an opportunity to share freely and surface themes that might not have been apparent a priori. While our findings may be informative for contexts similar to Singapore’s (densely populated urban settings, multi-generational households in a traditional hierarchical setting, or conflict-averse culture), they may be less generalizable to contexts where knowledge of the harms of SHS is better, smoking is still a norm, or where people are more comfortable with asserting their individual rights. As our study was conducted during the COVID-19 pandemic, our sample may under-represent certain groups, such as participants with limited online access.

## Conclusion

Residential SHS has wide-reaching negative impact on psychosocial wellbeing, especially in densely crowded settings where SHS is difficult to avoid. With no regulations covering smoking inside homes, neighbours are left to resolve the issue amongst themselves, often unsuccessfully due to various interpersonal, structural and cultural barriers.

## Supplementary Information

**Additional file 1.** Codebook with categories (in bold), themes, sub-themes, number of interviewees endorsing each theme, and sample quotations. *KI* Key informant, *NS* Non-smoker, *S* Smoker, *SHS* Secondhand smoke.

## Data Availability

The data generated and/or analysed during the current study are not publicly available for personal data protection reasons but are available from the corresponding author on reasonable request.
