# Supplementary material for: Residential secondhand smoke in a densely populated urban setting: a qualitative exploration of psychosocial impacts, views and experiences
Source: BMC Public Health. 2022 Jun 11;22:1168. doi: 10.1186/s12889-022-13561-7 (PMC9187883; doi:10.1186/s12889-022-13561-7)
Supplement: Supplementary file 1 — Additional file 1. Codebook with categories (in bold), themes, sub-themes, number of interviewees endorsing each theme, and sample quotations. KI Key informant, NS Non-smoker, S Smoker, SHS Secondhand smoke. [file 12889_2022_13561_MOESM1_ESM.docx]

Codebook with categories (in bold), themes, sub-themes, number of interviewees endorsing each theme, and sample quotations. KI=key informant; NS=non-smoker; S=smoker; SHS=secondhand smoke.

| **Theme** | **Sub-theme** | **KI** | **S** | **NS** | **Sample Quotations** |
| --- | --- | --- | --- | --- | --- |
| **Perceptions and experiences with SHS** | | | | | |
| Harm perceptions of SHS | Serious health issue | 6 | 13 | 13 | “…secondhand smoke is more toxic and more harmful than the firsthand smoke…In 2016, there were 2278 deaths [in Singapore] due to both active smoking and secondhand smoke, of which one out of six, 383 deaths were due to secondhand smoke.” P1 |
|  | Low but prolonged SHS harmful | - | 5 | 3 | “…I feel like over a long period of time, any exposure to secondhand smoking could be detrimental.” R24S  “Yeah, you don't see the immediate impact, but then the prolonged impact needs to be put into consideration.” R13N |
|  | Low SHS not serious | 3 | 9 | 1 | “That kind of low level, I guess is OK, I guess it's not so bad. Because your lungs…rest and doesn’t collect all these particles all the time.” R17S |
|  | Harm depends on other factors | - | 2 | 2 | “I'm sure if someone with asthma or some respiratory issues, it would be different. Smaller amounts could be an immediate reaction and all of that, but like seeing regular, healthy able-bodied, and stuff, I don't imagine it's that bad or maybe I'm just being hopeful.” R21Sb |
|  | Evidence on dose-effect | 2 | 1 | - | “… there is a need to show in concrete terms the harm that SHS poses… For example, exactly when, and at what levels and how, does exposure to SHS become harmful?” REG1 |
| In-home SHS from neighbours | Common | 8 | - | 1 | “Secondhand smoking can travel as far as 9 meters. One person's smoke can affect many units, upstairs, downstairs, and to the left and to the right.” AD1a |
|  | Personal experiences | 5 | 2 | 12 | “I've lived in three different condos in Singapore…In all three, I had smoker neighbours. It's not like moving house can solve this problem. Right now it's worse in this current condo that I'm staying in. We were sandwiched among three smokers, upstairs, downstairs and next-door.” AD1a |
|  | Hard to escape | 2 | - | 3 | “We go outside, we see people smoking from far, we know to avoid those. But at home, it's very difficult for us to avoid these kind of things.” R14N |
| SHS in common residential areas | Personal experiences | 2 | 11 | 8 | “As long as you don't get caught, it's not a problem. So I have seen people smoking at the void decks, I have seen people smoking in the corridors… we do it, but we do it discreetly.” R19S |
|  | Enforcement | 3 | 3 | - | “I saw someone at my void deck, got caught by an NEA officer.” R19S |
| SHS exposure following the COVID-19 pandemic | Worsened | 9 | 1 | 10 | “When the lockdown began, I began to smell quite a lot of secondhand smoke. I never had this problem before the lockdown, and then it intensified in the middle of the year, of 2020...” AD2 |
|  | Improved or same | - | 2 | 4 | “I think it was better during Circuit Breaker. Because he had to stay in [the Army] camp.” R5N  “… he's a taxi driver, so he goes everywhere, even during circuit breaker he's still working, so nothing has changed for him.” R9N |
| Personal impacts of SHS | Physical health | 6 | 3 | 8 | “She [neighbour] has a toddler. The poor toddler has been suffering from chronic bronchitis and even pneumonia due to her downstairs chain-smoker neighbour.” AD1b |
|  | Mental wellbeing | 6 | - | 5 | “…my wife is so stressed, she can’t sleep. Every night she’s got to check on the children, see whether they’re okay.” AD3 |
|  | Discomfort | 2 | 2 | 13 | [On having to keep doors closed most of the time:] “…my toilet floor is always wet and because when it's wet and it's moist, it affects my walls and windows with mould and mildew and all this kind of stuff.” R12N  “I can't even smell fresh air in my own personal space.” R22N |
|  | Smokers dislike SHS | 2 | 8 | 1 | “as a smoker, I hate secondhand smoke. I don't like the smell.” R17S |
|  | Financial impact | 1 | 1 | 3 | “It’s a lot of costs on our side as well. It's not free, to just wash clothes. You have to buy detergent, the water supply is paid for. And the more you wash with, the more water use, the more the water bills go up.” R14N |
| **Minimising SHS from neighbours** | | | | | |
| Strategies to minimise SHS from neighbours | Doors and windows | 1 | - | 13 | “So closing the windows is the most fool-proof way to prevent the smoke from coming in, at the cost of stuffiness and lack of ventilation.” R4N |
|  | Air purifier or fan | 2 | 3 | 4 | “I've tried air purifier before. It doesn't work because it's not fast enough.” R4N |
|  | Move to another area | - | - | 2 | “So, the moment I detect the smoke I quickly tell them [children], hey, there’s somebody smoking, you all go to your room, close the door, or go to the living room.” R12N |
| Confronting neighbours about SHS | Friendly approach | 5 | - | 1 | “And I would try to just casually get into a chat and understand what's the habit, why you're doing certain things. Is there alternatives? So kind of, make suggestions to them, if saying sometimes, you know, people work around here or they get very young children, your neighbour has some medical condition, asthma or other medical conditions, this will cause a lot of inconvenience to them.” P2 |
|  | Antagonistic approach | 1 | - | 4 | “…she will go upstairs and like scold the person and be like “look at my clean clothes now.” R11N  “…a fellow resident in the block typed out their own message and pasted it in the lobby.” R15N |
|  | Fear of conflict | 5 | 2 | 8 | “I don't want to actually confront them because that would put me in a difficult position… what if the person gets aggressive?” R12N |
|  | No legal grounds | 5 | - | 6 | “…with no regulation for smoking in the house, there’s technically nothing that we as neighbours who are non-smokers can do about it.” R14N |
| Smokers’ responses to neighbour confrontation | Reluctant or hostile | 7 | 4 | 7 | “We try to approach all three [smoking neighbours], but to no avail. The downstairs smoker refused to open the door on multiple occasions. The upstairs smoker insisted that it's his right to smoke at home, because it's not against the law and told us to mind our own business. Then the next-door neighbour turned aggressive...” AD1a  “I will get a bit defensive because this is my house…” R19S |
|  | Open to change | 2 | 13 | 4 | “…we don't want any trouble with our neighbours. We have a very good relationship with our neighbours.” R26S |
|  | Depends on neighbour | - | 7 | - | “It really depends on how belligerent they are about it, to be honest… If someone's bringing up health complications, then I would be a lot more understanding and, of course, sympathetic.” R21Sa |
|  | Protecting family | 5 | 3 | 3 | “…he’ll run to the kitchen window and smoke, which I think is not nice to the other neighbours but you can see he’s trying to be considerate for his grandchildren and his guests...” R14N |
|  | Mobility issues | - | - | 1 | “…he’s got some mobility issues, some health condition… for him specifically to go down and smoke and taking our time, I think it’s a bit, it’s pretty hard for us. That’s why we allow him to smoke in the house instead.” R16N |
| **Minimising SHS from smokers in the household** | | | | | |
| Smokers’ strategies to minimise SHS | Smoke in other areas | - | 8 | 3 | “We have a smoking corner in the kitchen, that's like the communal area, but most of the time, like for my brother and I, we just do it in the room, our separate rooms, because we are cooped up in our own rooms.” R27S |
|  | Smoke out the window | - | 6 | 2 | “I would make sure that I opened a window and whenever I’m smoking I’m facing the fan towards the window so it blows out.” R24S |
|  | Close doors or windows | - | 3 | 4 | “There's this tiny room that's attached to the toilet. There's actually a door, so usually whenever he smokes he'll close that door so the whole house doesn't smell like cigarettes.” R24S |
|  | Smoke when alone | - | 1 | 1 | “he tends not to smoke when we are around. So when we’re not around, I’m either working or mother step out of the kitchen for a while then he will tend to smoke.” R16N |
| Non-smokers’ strategies to minimise SHS | Doors and windows | - | - | 8 | “I also put things to block the gap between the door and the floor, to make sure no air comes into the room.” R10N |
|  | Avoid area | - | 1 | 4 | “I got fed up, I spend all my time in the room.” R8N |
|  | Air purifier or fan | - | 3 | 6 | “It’s an attempt but it’s not exactly useful.” R10N |
| Confronting smokers about SHS in the home | Ask to smoke elsewhere | - | - | 5 | “…we told him not to smoke anywhere, to just use the kitchen window in the service area.” R16N |
|  | Ask to quit | - | 3 | 6 | “I don’t think I have tried to get him to quit for the benefit of his self. It's more like for me because I'm affected” R5N |
|  | Personal health risk | - | - | 7 | “…I use my mom’s illness for example to say that later I become a secondhand smoker then I will have lung cancer.” R6N |
|  | Express discomfort | - | - | 7 | “I’m very angry, I scream at him.” R8N  “…like being sarcastic, like cough in front of them when they smoke…” R22N |
|  | Willing to change | - | 1 | 8 | “He used to smoke indoors… Then, yeah, we just kept, I guess, nagging at him and annoying him until he goes out...” R11N |
|  | Reluctant to change | - | - | 9 | “…I always say, “can you at least do it outside?” But he'll just be like, yah lah, ya lah, ya lah. But he doesn't do it. Yeah.” R5N |
| Issues in addressing in-home SHS | Guilt or conflict | - | 3 | 3 | “I get anger that, wah, you just don’t bother, you’re just enjoying yourself smoking. You don’t care about me, a non-smoker, inhaling all this.” R8N |
|  | Convenience | - | - | 3 | “I think why he smokes in the room is because of convenience.” R5N |
|  | Ingrained habit | - | - | 6 | “…the other day he said to give him a break because he's trying very hard already.” R2N |
|  | Traditional norms | 1 | 3 | 5 | “You don't own the house. You do not dictate to him [what to do]. I mean, he has, you know, Asian society, he’s still the senior.” R2N |
| **Views on socially responsible smoking** | | | | | |
| Views on socially responsible smoking | Entitlement | 5 | 6 | 4 | “…it’s my house, I should be able to do what I want to do inside my house.” R7S |
|  | No right to affect others | 9 | 4 | 4 | “…Neighbours or people who live in their house and say, “it’s my house, my own problem, I smoke, my own problem. I don’t disturb you”, which is a very wrong concept they have, because definitely they’re bothering somebody.” R14N |
|  | Responsible smokers | 3 | 10 | 3 | “When we just moved in… I also told my neighbour up front that if the cigarette smoke is impacting them please let me know and I will find another corner or just go downstairs to smoke.” R20S |
|  | Irresponsible smokers | 6 | 4 | 7 | “I think people tend to look out for themselves only. When they smoke they just want to shut the urge for that moment. They don't realize how it could affect others.” R23S |
|  | Selfish culture | 3 | - | 1 | “…we’re just generally very entitled people. We don’t really care about other people.” R4N |
